# Oligometastatic colorectal adenocarcinoma to the spleen and ovaries

**DOI:** 10.1093/jscr/rjae241

**Published:** 2024-04-18

**Authors:** Lucienne Heath, Elan Novis, Joel Rabindran, Alexander van Laar Veth, Tao Yang, Megan B Barnet, Rohan Gett

**Affiliations:** Department of General Surgery, St Vincent’s Hospital, 390 Victoria St, Darlinghurst, NSW 2010, Australia; Department of General Surgery, St Vincent’s Hospital, 390 Victoria St, Darlinghurst, NSW 2010, Australia; Department of General Surgery, St Vincent’s Clinical School, University of New South Wales, 390 Victoria St, Darlinghurst, NSW 2010, Australia; Department of General Surgery, St Vincent’s Hospital, 390 Victoria St, Darlinghurst, NSW 2010, Australia; Department of General Surgery, St Vincent’s Clinical School, University of New South Wales, 390 Victoria St, Darlinghurst, NSW 2010, Australia; Department of Anatomical Pathology, St Vincent’s Hospital, 390 Victoria St, Darlinghurst, NSW 2010, Australia; Department of Anatomical Pathology, St Vincent’s Hospital, 390 Victoria St, Darlinghurst, NSW 2010, Australia; Department of General Surgery, St Vincent’s Clinical School, University of New South Wales, 390 Victoria St, Darlinghurst, NSW 2010, Australia; Department of Medical Oncology, Garvan Institute of Medical Research, 384 Victoria St, Darlinghurst, NSW 2010, Australia; School of Biomedical Engineering, University of Technology Sydney, 11/81 Broadway Ultimo, NSW 2007, Australia; Department of General Surgery, St Vincent’s Hospital, 390 Victoria St, Darlinghurst, NSW 2010, Australia; Department of General Surgery, St Vincent’s Clinical School, University of New South Wales, 390 Victoria St, Darlinghurst, NSW 2010, Australia; Department of General Surgery, St Vincent’s Private Hospital, 406 Victoria St, Darlinghurst, NSW 2010, Australia

**Keywords:** splenic metastasis, ovarian metastasis, colorectal cancer

## Abstract

In the context of colorectal cancer, splenic and ovarian metastases are rare outside of widely disseminated disease. Growing evidence suggests that ‘oligometastatic’ or limited metastatic disease can be treated surgically with good oncological outcomes. Splenic and ovarian metastases are not well represented in studies of oligometastatic colorectal cancer, resulting in uncertainty in the best management for these patients. We present the case of a 78-year-old woman diagnosed with oligometastatic colorectal cancer to bilateral ovaries and spleen, 5 years after resection of a primary colon cancer. The patient was treated with a bilateral salpingo-oopherectomy and subsequent open splenectomy. We discuss the role of surgery and peri-operative chemotherapy in the management of oligometastatic colorectal cancer involving atypical sites.

## Introduction

Colorectal cancer is the third most commonly diagnosed cancer in Australia and the second most common cause of cancer-related death [1]. Approximately 20% of patients are diagnosed with *de novo* metastatic disease, and >30% of patients with resected early disease will recur within 5 years [2, 3]. Stage at diagnosis remains the most important prognostic factor, with unresectable Stage IV colorectal cancer conferring a 5-year survival of just 13% [4]. ‘Oligometastatic’ disease, defined here as less than five lesions at up to two sites, can be treated surgically with long-term survival in up to 45% of patients [5].

The most common sites for colorectal cancer recurrence are liver and lung, which are involved in 70% and 30%–50% of cases, respectively [6]. Splenic metastases predominantly occur in the context of disseminated disease involving liver (77%) or lung (36%) [7]. Ovarian metastases are uncommon, occurring in 2.1%–13.6% of patients following colorectal resection, and usually in the context of disseminated peritoneal disease [8]. Here we present a case of oligometastatic disease to spleen and bilateral ovaries, presenting 5 years after resection of a primary descending colon cancer.

## Case report

A 78-year-old woman presented with symptoms of a large bowel obstruction and was found to have a large splenic flexure tumour. She was otherwise well, with a history of asthma, hypertension and hysterectomy for fibroids with ovaries *in situ*. She underwent a subtotal colectomy. Pathology showed a pT4aN2 colon adenocarcinoma, with perineural invasion, 4 of 59 nodes involved, proficient mismatch repair proteins and BRAF-wild-type. She recovered well post-operatively and received adjuvant chemotherapy with combination fluorouracil and oxaliplatin, truncated at 3 months’ due to toxicity.

**Figure 1:**
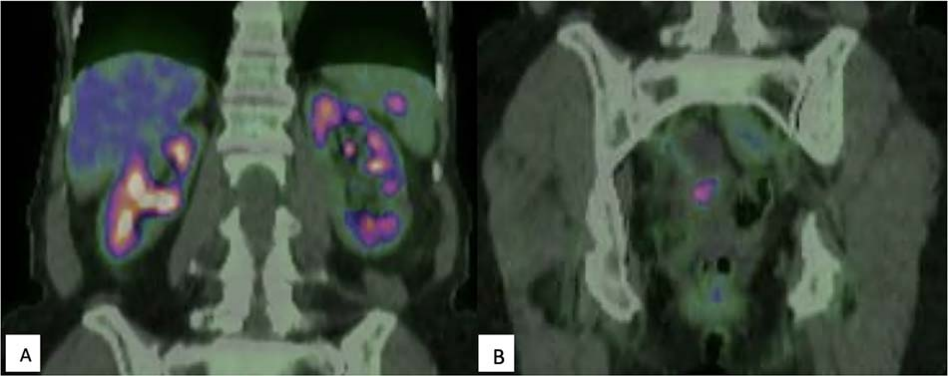
(A) FDG-PET demonstrating moderately intense (SUVmax 5.6) uptake within the spleen, suspicious for malignancy; (B) Multicystic pelvic lesion with solid component (SUVmax 4.0).

Surveillance CT 5 years after the completion of chemotherapy demonstrated bilateral pelvic adnexal ‘cysts’. These were investigated further with pelvic ultrasound and FDG PET, which demonstrated the large cystic lesions to have foci of moderate FDG avidity (SUVmax 4.0), with incidental finding of a moderately FDG-avid (SUVmax 5.6) lesion at the hilum of her spleen (Fig. 1).

Following multi-disciplinary discussion, a bilateral salpingo-oopherectomy was performed for diagnostic clarity and ongoing pelvic discomfort. Histopathology confirmed bilateral ovarian metastatic adenocarcinoma consistent with colorectal primary. The patient recovered well and was planned for subsequent splenectomy.

Endoscopic examination of the rectum and ileorectal anastomosis did not demonstrate any evidence of recurrence or metachronous lesions. The patient underwent a routine open splenectomy and made an uneventful recovery. Histopathology demonstrated three circumscribed, firm and pale metastatic adenocarcinoma deposits within the splenic parenchyma, 5–10 mm in maximal dimension.

**Figure 2:**
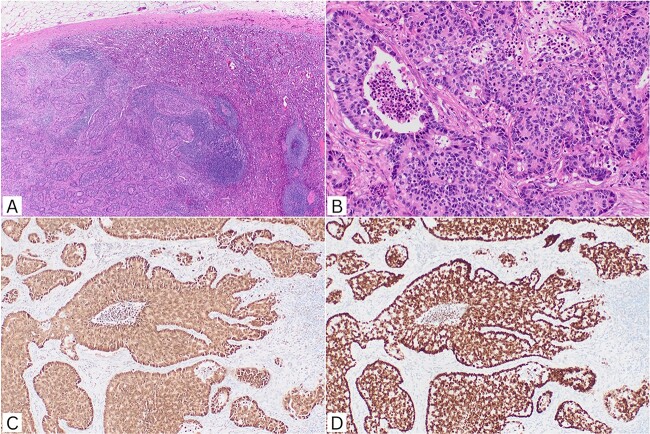
Histopathological features. (A) Metastatic adenocarcinoma infiltrating splenic parenchyma (H&E, ×40). (B) Irregular cribriform glands with luminal necrosis in keeping with colorectal origin (H&E, ×200). (C) CDX2 nuclear positivity (IHC, ×100); (D) SATB2 nuclear positivity (IHC, ×100). H&E: haematoxylin and eosin; IHC: immunohistochemistry.

Morphology and immunohistochemistry was consistent with colorectal origin. The metastatic deposit of adenocarcinoma within the splenic parenchyma demonstrated poorly formed and cribriform glands lined by crowded columnar cells with central luminal necrosis. There was no abnormality in the background spleen. The immunohistochemical profile confirmed colorectal origin with tumour cells positive for CK20, CDX2, and SATB2. The tumour was negative for CK7, TTF-1, ER, GATA3, PAX8, synaptophysin, and chromogranin A (Fig. 2).

At 6 months post-splenectomy and 9 months post-bilateral salpingo-oopherectomy, the patient was at baseline excellent functional status with a CEA of 3.8 and no evidence of recurrence on FDG-PET imaging. No further systemic therapy was prescribed after multi-disciplinary discussion due to previous poor tolerance and no evidence of survival benefit for chemotherapy post colorectal cancer metastatectomy [9]. She was re-engaged in a close surveillance protocol.

## Discussion

This case demonstrates a rare case of isolated splenic and ovarian oligometastases from a colorectal primary, 5 years after resection of the initial primary. This is a highly unusual occurrence in the absence of widespread metastatic disease. The patient had a good early outcome to treatment with surgical resection alone, however long-term outcomes are not yet known.

Oligometastatic cancer represents a poorly defined clinical state between localized and widespread disease [10]. Within colorectal cancer, oligo-metastatic disease confined to liver and/or lung is well studied [6]. Optimal management usually includes multi-disciplinary discussion of local therapy options, with consideration of patient and tumour factors [11]. For extra-hepato-pulmonary metastases the roles of resection, neoadjuvant and adjuvant chemotherapy is less clear.

Several small, retrospective studies have examined metastectomy for concurrent liver and lung lesions. Shah et al. demonstrated improved 5 year survival of 74% in those who had both liver and lung metastases resected, compared to 42% in those who received liver resection only [12]. Wei *et al.* [13], in their prospective trial, highlight the significant morbidity and mortality associated with such invasive disease with one perioperative death out of 26 patients and a major complication rate of 19%. The SABR-Comet trial found benefit for stereotactic radiotherapy in oligometastatic disease across mixed tumour-types, supporting non-surgical approaches where clinically appropriate [14].

The spleen is thought to have both an inhibitory effect of immune cells on the deposition of tumour cells and haematogenous spread limited by anatomic blood flow, resulting in an overall low likelihood of metastases [7, 15].There are just 37 case reports of isolated splenic metastases from colorectal cancer, which have almost exclusively undergone splenectomy by laparoscopic or open approach [7]. The longest follow-up without recurrence documented is 72 months [16]. Thirteen cases report their follow-up period, and two patient mortalities secondary to widespread metastases [7]. Chemotherapy was specified in 11 cases, 1 neoadjuvant and 10 adjuvant [7].

Ovarian metastases have traditionally been thought of as a poor prognostic factor [17], however a more recent case series found R0 resection of isolated ovarian metastases had a longer 5 year survival rate at 69% than isolated hepatic metastases and isolated pulmonary metastases at 42%–60% and 51%–63% [8].

This limited evidence highlights the difficulty in selecting patients for significant intra-abdominal surgery where recurrence is limited. Potential for improved outcomes with aggressive management of oligometastatic colorectal cancer to liver and/or lung is well documented, however, appropriate management of disease at atypical sites is less clearly defined. Our patient had bilateral salpingo-oopherectomy for suspicious adnexal lesions >5 years post her initial colorectal cancer resection, and proceeded to open splenectomy with curative intent. This was supported by the prolonged time to recurrence, and absence of nodal or peritoneal disease. Multidisciplinary discussion was invaluable, and is suggested for all patients with oligometastatic disease regardless of unusual location.
